# The Antibiotics Used in Livestock and Their Impact on Resistance in *Enterococcus faecium* and *Enterococcus hirae* on Farms in Gabon

**DOI:** 10.3390/antibiotics11020224

**Published:** 2022-02-10

**Authors:** Désiré Otsaghe Ekore, Richard Onanga, Pierre Phillipe Mbehang Nguema, Chloé Lozano, Brice Serge Kumulungui

**Affiliations:** 1 Centre International de Recherche Médicales de Franceville, Franceville, Gabon; onangar@yahoo.fr (R.O.); mbehangphilippe@gmail.com (P.P.M.N.); chloelozano@protonmail.com (C.L.); kumulungui@yahoo.fr (B.S.K.); 2 Ecole Doctorale Régional d’Afrique Central, Franceville, Gabon; 3 Institut de Recherche en Ecologie Tropical, Libreville, Gabon

**Keywords:** *E. faecium*, *E. hirae*, antibiotics, livestock, Gabon

## Abstract

The emergence of antibiotic resistance is a major concern around the world. The objective of this study was to investigate the antibiotics used in livestock and their impact on resistance in *Enterococcus faecium* and *Enterococcus hirae* on farms in Gabon. A structured questionnaire was used to collect information on the farms. Samples were collected from farms (*n* = 20) tested for *Enterococcus* by culture and isolation and were identified using a polymerase chain reaction (PCR) and sequencing. Antibiotic susceptibility was determined by the disc diffusion method on Mueller Hinton agar. The 20 farms included laying hens (6), swine (6), sheep (4) and cattle farms (4). Tetracycline was the most used antibiotic family (91%) and the most used prophylactic method (47%) for the treatment of animals. A total of 555 samples were collected and 515 (93%) *Enterococcus* spp. isolates of the genus were obtained. The prevalence of *E. faecium* and *E. hirae* were 10% and 8%, respectively. The isolates from *E. faecium* and *E. hirae* we found were related to clinical and human isolates in the NCBI database. *E. faecium* and *E. hirae* isolates showed a high resistance to tetracycline (69% and 65%) and rifampicin (39% and 56%). The *tet(M)* gene was detected in 65 tetracycline-resistant isolates with a large majority in hens (78% (21/27) and 86% (12/14) in *E. faecium* and *E. hirae*, respectively). The consumption of antibiotics favours the emergence of antibiotic resistance in animals in Gabon.

## 1. Introduction

*Enterococcus* contains commensal and opportunistic bacteria found in humans, animals and the environment [1,2]. It is used as an indicator of faecal contamination in food products from animals [3,4]. In humans, *Enterococcus* is an important opportunistic pathogen with *E. faecalis* and *E. faecium* being implicated in infections in hospital settings [5]. Its persistence in the environment and the plasticity of its genome allow *Enterococcus* to acquire antibiotic resistance genes and to colonise several ecological niches [5]. *Enterococcus* species have an intrinsic resistance to aminoglycosides (a low-level resistance), penicillins, vancomycins (*E. gallinarum*, *E.* *casseliflavus*), polymyxins and streptogramins [6]. *E. durans*, *E. hirae*, *E. gallinarum*, *E. casseliflavus*, *E. faecalis* and *E. faecium* are often found in the digestive tract of farm animals [6,7]. The presence of others resistances in *Enterococcus* species could be the result of antibiotic use on farm animals.

The consumption of antibiotics by livestock has increased in recent decades due to the increasing demand for animal protein [8]. In the livestock sector, antibiotics are used for prophylaxis, therapeutics, metaphylaxis and as growth promoters to keep animals healthy and ensure a high production [9]. However, their misuse could lead to the emergence of antibiotic-resistant bacteria in these animals and thus create reservoirs of resistance genes [10,11]. These resistance genes are potentially transmitted to humans through direct or indirect contact [12,13]. Therefore, studies of antibiotic consumption are needed to control and prevent misuse in livestock.

In Gabon, studies have characterised the phenotypic and genotypic resistance in terrestrial mammals [14,15], the chicken meat trade [16] and hospital settings [17,18]. These studies showed high rates of resistance to chloramphenicol, ampicillin and gentamicin in humans [18] and to tetracycline, ampicillin and cephalosporin in bats [19] and commercial hens [16]. However, no studies have been conducted to characterise resistance in farm animals. Thus, the objective of our study was to investigate the antibiotics used on livestock and their impact on *E. faecium* and *E. hirae* resistance on farms in Gabon.

## 2. Results

### 2.1. Number of Faecal Samples Collected

Five hundred and fifty-five faecal samples were collected from livestock in seven Gabonese provinces including laying hens (*n* = 209), swine (*n* = 196), cattle (*n* = 69) and sheep (*n* = 81). The sample size for each animal species was significantly representative of the population collected in this study (χ^2^ = 157.72; *p* < 0.05).

### 2.2. Characteristics of the Study Population

The 20 farms were characterised according to the animal species: 6 (30%) laying hen farms, 6 (30%) swine farms, 4 (20%) sheep farms and 4 (20%) cattle farms. The livestock on these farms consisted of 14 (70%) exotic breeds, 5 (25%) local breeds and 1 (5%) mixed breed. The proportion of exotic breeds was significantly higher than of local breeds (χ^2^ = 19.95; *p* < 0.05). The livestock consisted of 13 (65%) intensive livestock, 4 (20%) semi-intensive and 3 (15%) unspecified. The proportion of intensive farming was significantly higher than the semi-intensive farming (χ^2^ = 13.65; *p* < 0.05).

### 2.3. Drug Use on Livestock

Of the 20 farmers, 15 answered the questions and 5 did not. The “no response” category included farmers who did not respond to the questionnaire or whose questionnaire was incomplete. The treatment methods used by the farmers were prophylaxis (*n* = 7, 47%), therapeutic (*n* = 5, 33%) and prophylaxis therapy (*n* = 3, 20%). Of the 15 questionnaires completed, 11 farmers used antibiotics on their livestock, of whom 10 (91%) used tetracycline, three (27%) polymyxin (colistin) and two (18%) ampicillin. There was a significant difference of proportion between tetracycline and the other antibiotics used to treat the animals (χ^2^ = 10.13; *p* < 0.05).

### 2.4. Distribution of E. faecium and E. hirae

A total of 515/555 (93%) isolates of *Enterococcus* spp. were obtained. The prevalence of *E. faecium* was 10% (*n* = 54) and that of *E. hirae* was 8% (*n* = 43). *E. faecium* was found in 32/209 (15%) faecal samples from laying hens, 17/196 (9%) from swine, 4/81 (5%) from sheep and 1/69 (1%) from cattle. *E. hirae* was found in 18 (9%) samples from laying hens, 15 (8%) from swine, 6 (8%) from sheep and 4 (6%) from cattle.

### 2.5. Phylogeny of E. faecium and E. hirae from Animals

**Figure 1:**
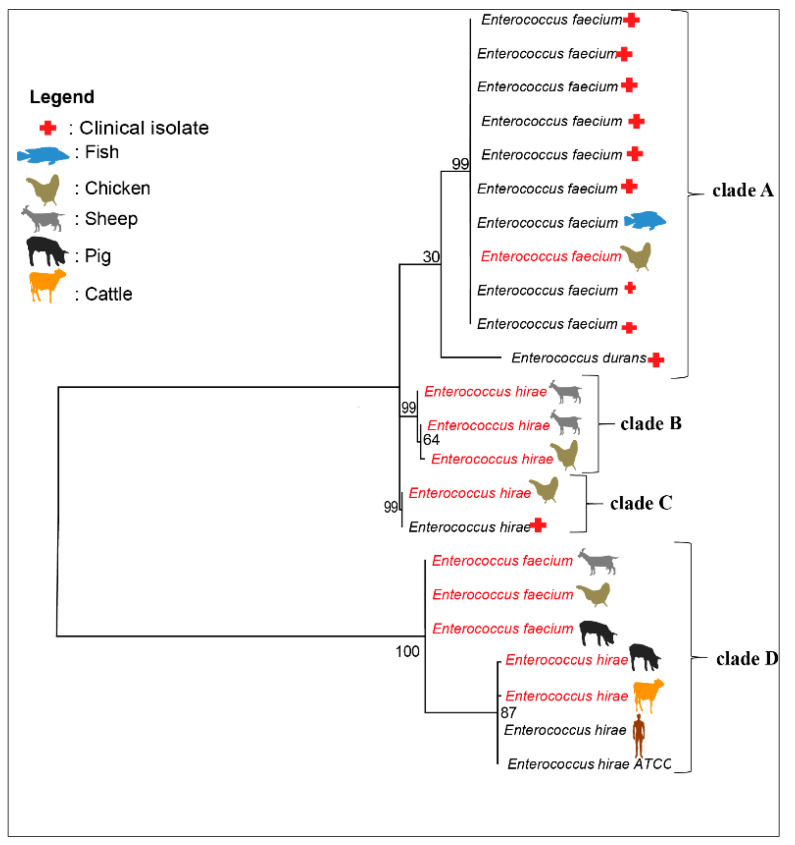
Phylogenetic analysis of *E. faecium* and *E. hirae*. The phylogenetic tree was constructed by a sequence homology of the *sodA* gene between four *E. faecium* and six *E. hirae* isolates randomly (black) selected from our study and those in from the NCBI database (red).

The phylogenetic tree of the *sodA* gene showed 4 clades (Figure 1). These clades were grouped on the basis of the sequence homology between four *E. faecium* (chicken, sheep and pig) and six *E. hirae* (chicken, sheep, pig and cattle) isolates randomly selected from 97 samples obtained in the current study and the NCBI database from clinical, human and fish isolates (Figure 1). In clade A, an *E. faecium* isolate from laying hens clustered with clinical *E. faecium* strains (CP039729.1, CP046077.1) and a fish isolate (CP045012.1). Clades B and C were composed exclusively of three *E. hirae* and *E. faecium* isolates obtained in this study. In clade D, two *E. hirae* isolates from swine and cattle in this study formed a cluster with a human isolate (*E. hirae* strain 708) and a reference *E. hirae* strain ATCC 9790. However, there were many other clades that were not shown (if other NCBI isolates were selected).

### 2.6. Antibiotic Susceptibility Profile of E. faecium and E. hirae

Of the 54 isolates of *E. faecium*, resistance was very high for tetracycline (69% (37/54)) and high for rifampicin (39% (21/54)) and vancomycin (37% (20/54)). Resistance to teicoplanin was very low (6%, 3/54). The *E. hirae* isolates showed a high resistance to tetracycline (65%, 28/43) followed by rifampicin (56%, 24/43) and vancomycin (19%, 8/43) and a low resistance to teicoplanin (2.3%, 1/43).

### 2.7. Distribution of Antibiotic Susceptibility by Animal Species

*E. faecium* and *E. hirae* showed a diversity of resistance to antibiotics among the livestock species. *E. faecium* from the laying hen isolates showed a high resistance to tetracycline (84%), rifampicin (34%) and vancomycin (22%) whereas resistance to teicoplanin was very low (6%). The swine isolates showed a high resistance to vancomycin (76%), rifampicin (76%) and tetracycline (59%). Resistance to teicoplanin was very low (6%) in swine. The only resistance found in sheep was rifampicin (75%). No resistance was observed in cattle.

For *E. hirae*, the laying hen isolates showed a high resistance to tetracycline (78%) followed by rifampicin (34%) but a low resistance to vancomycin (6%) and no resistance to teicoplanin. The swine isolates showed a high resistance to tetracycline (73%) and rifampicin (67%) but a low resistance to vancomycin (20%) and a very low resistance to teicoplanin (7%). Among the cattle isolates, a high resistance was observed to vancomycin (75%) and rifampicin (75%) but there was no resistance to tetracycline or teicoplanin. The sheep isolates showed a high resistance to rifampicin (83%) and tetracycline (50%), a low resistance to vancomycin (17%) and no resistance to teicoplanin.

### 2.8. Characterisation of the tet(M) Gene

**Figure 2:**
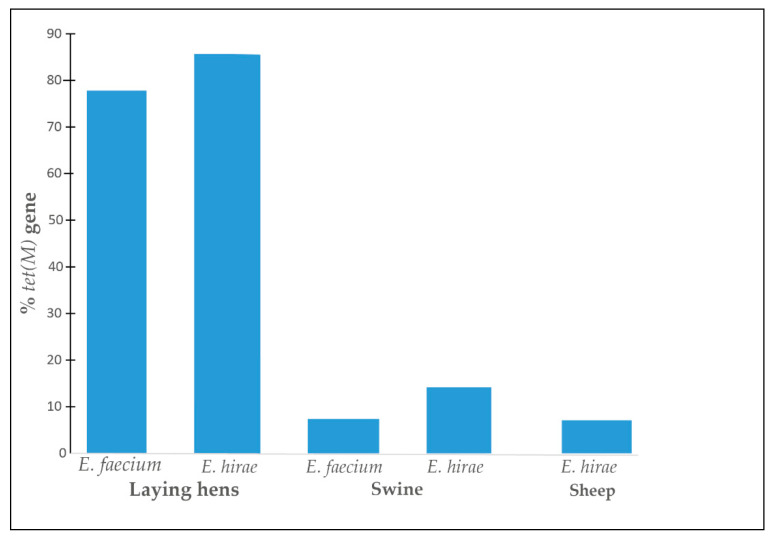
Prevalence of the *tet(M)* gene found in *E. faecium* and *E. hirae* isolates in animal species.

We found the *tet(M)* gene for 65 tetracycline-resistant isolates in our study. Laying hen isolates showed a high prevalence of this gene in *E. faecium* (78%, 21/27) and *E. hirae* (86%, 12/14) but the prevalence was low in the swine isolates (7%, 2/27 for *E. faecium* and 14% (2/14) for *E. hirae*). In sheep, the *tet(M)* gene was found in one *E. hirae* isolate (7%, 1/14). No tetracycline-resistant isolates were identified in cattle (Figure 2).

## 3. Discussion

Antibiotic resistance is a major problem in the world today in the environment, animals and humans. The emergence of resistance in livestock is most often the result of antibiotic consumption [20]. Studies of the use of antibiotics and their resistance in livestock production are important to better understand the emergence and spread of resistance. In this study, we described antibiotics used in livestock and antibiotic resistance in *E. faecium* and *E. hirae* on farms in Gabon.

Our sample included laying hen, swine, sheep and cattle farms. These species are the most heavily exploited in Gabon [21]. The intensive breeding of exotic breeds was most common on the farms. These results are similar to those found in studies conducted in Nigeria (62%) [22]. However, intensive breeding favours the development and spread of pathogens in livestock and, potentially, transmission to humans [23]. This could be explained by the fact that the farm environment can carry pathogens that can be transmitted to a few animals and then spread to other animals on the same farm [24,25]. Exotic breeds are much more profitable in terms of production but are often subject to diseases, most of which need to be treated with drugs thus leading to a lower productivity [26].

Treatment methods are very useful for the prevention and treatment of diseases that can affect livestock. Our study found that prophylactic (47%) and therapeutic methods (33%) were used to treat the animals on farms. Similar results were reported in Bangladesh [27] where the prophylactic method, the therapeutic method and both methods simultaneously were used on farms. Prophylactic and therapeutic methods use different antibiotics to treat animals, selecting the optimal agent based on effectiveness. In our study, tetracycline (91%) was the most commonly used antibiotic on farms. Our results were similar to those reported for Nigeria (90.8%) [22] and Tanzania [20] (62.9%). Oxytetracycline, an antibiotic of the tetracycline family, is widely used in the veterinary industry for its broad spectrum activity as it allows the elimination of a large number of pathogens [28,29]. Its high use in livestock could be related to its lower cost as well as its availability without prescription from a veterinarian. However, the inappropriate use of antibiotics for the treatment or prevention of disease could be the cause of the emergence and dissemination of resistance genes in the food chain that could potentially be transmitted to humans.

The distribution of the enterococcal species in our study showed a prevalence of 10% for *E. faecium* and 8% for *E. hirae* in farm animals. Klibi et al. [30] observed *E. faecium* (25%) and *E. hirae* (10%) mostly in meat samples (poultry, beef and sheep) in Tunisia. Iweribor et al. [31] also characterised *E. faecium* (35%) and *E. hirae* (31%) as the most isolated species among pigs in South Africa. These two species are important because of their ability to acquire resistance genes and disseminate them in the intestinal tract [4]. This makes them a good marker for the evaluation of antibiotic resistance in livestock. Furthermore, *E. faecium* and *E. hirae* in the animals studied were related to clinical strains extracted from the NCBI database. Freitas et al. [32] recently showed a link between food-derived and clinical isolates of *E. faecium*. Other similar studies have also shown relationships between the clinical and animal isolates of *E. faecium* [33,34]. This highlights the zoonotic potential of the isolates from farm animals. The zoonotic transfer of pathogens from animals to humans is possible through direct or indirect contact and may be responsible for the dissemination or acquisition of antibiotic resistance genes.

In our samples, a very high prevalence of tetracycline resistance was found among the *E. faecium* and *E. hirae* species. This prevalence, which was particularly high in laying hens and pigs, was similar to those observed in chickens in Angola (66.6%) [35] and Nigeria (81.6%) [36]. This result was also consistent with the high use of tetracycline on farms in our survey. The high prevalence of tetracycline is likely related to the consumption of this antibiotic by livestock.

The rifampicin resistance phenotypes obtained from the cattle (75%) and sheep (83.3%) isolates were comparable with those found in Tanzanian livestock (63% and 72% rifampicin resistance, respectively) [37,38,39] and Nigeria (90% in horses) [40]. This observation was surprising because this broad spectrum antibiotic was not described in the farms we surveyed. This antibiotic is only used for the treatment of tuberculosis-related mycobacterium infections in Gabon [41,42]. We therefore assumed that this resistance was acquired through the consumption of waste from humans or the environment. High rates of resistance to vancomycin were also obtained in our study (37% for *E. faecium* and 19% for *E. hirae*, respectively). This result was also surprising because vancomycin (or other glycopeptides) was not mentioned among the antibiotics used in the farms. We believe this to be a persistence of resistance due to the origin of exotic animals from Europe where a high prevalence of this resistance has been observed [43,44] due to the use of avoparcin as a growth promotor in livestock [45,46]. Under this hypothesis, the observed prevalence was the result of a horizontal transfer of resistance genes among these animals.

The *tet(M)* gene coding for ribosomal protection is the most frequently detected tetracycline resistance gene in cattle regardless of the origin of the isolates [6,36]. In our study, 65 *tet(M)* genes were described, mainly in laying hens. Klibi et al. [30] characterised a high prevalence of this gene (49%) from meat (poultry, sheep and beef) in Tunisia. Fazzon et al. [47] also characterised 38% of isolates of the *tet(M)* gene from food in Brazil. The presence of the *tet(M)* gene is most often associated with conjugative elements that are important factors in the spread of tetracycline resistance [36,47].

In summary, the very high rates of tetracycline resistance in our study were likely related to its consumption on farms whereas the high frequency of resistance to vancomycin and rifampicin was likely due to other sources of resistance acquisition such as human waste consumption or the environment.

## 4. Materials and Methods

Ethics statement: This study was conducted in Gabon and approved by the Gabonese Ministry of Agriculture, Livestock, Fishery and Rural Development (General Direction of Livestock, Authorisation n°0052/SG/DGE). All samples from the farm animals were collected after verbal consent was obtained from the managers of the farms.

Structure of the questionnaire: This was structured with open-ended questions on social data, animal health, hygiene, antibiotics consumed by animals, other medicines used on these farms, strategies used in the management of animal health, species and breeds of animals (a questionnaire table is provided in the Supplementary File S1).

**Figure 3:**
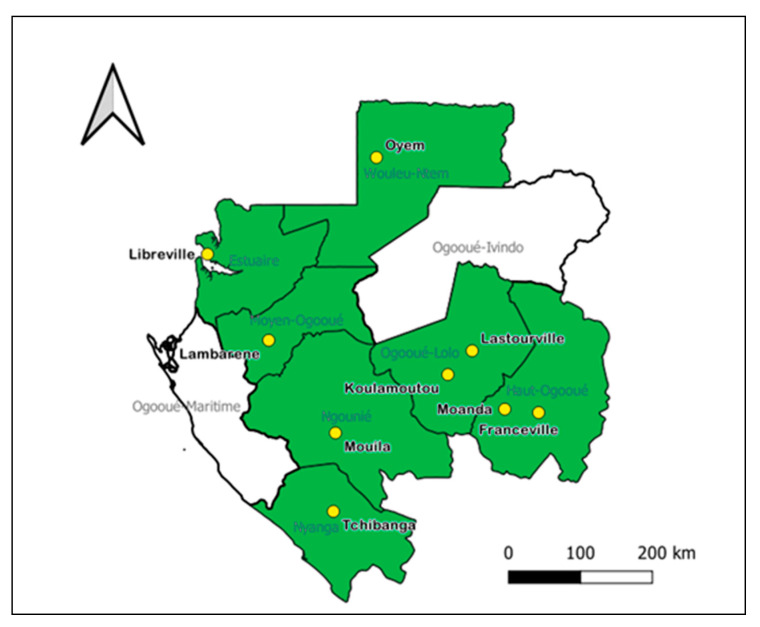
Sampling locations. Green indicates the provinces and yellow represents the cities where the animals were sampled.

Faecal sampling: Sampling was conducted on 20 farms from 7 provinces of Gabon (Estuaire, Haut-Ogooué, Moyen-Ogooué, Ngounié, Nyanga, Ogooué-Lolo, Woleu-Ntem) from December 2018 to January 2020 (Figure 3). We collected fresh droppings and rectal swabs. The capacity of the farms was distributed as follows: laying hens (901–1501 (1), 1001–1100 (1), 1701–1800 (1)); cattle (1–10 (2), 101–150 (1), 301–350 (1), 1001–1100 (1)); swine (11–20 (1), 41–60 (2),81–100 (1), 201–250 (1)); sheep (21–40 (2), 41–60 (1)). To avoid repeatability, 10 to 15% of the total population was sampled in the hens. Each faecal sample was collected in a sterile, plastic jar (Qualibacter, France), which we sealed hermetically and transported to the Centre International de Recherche Médicale de Franceville (CIRMF) bacteriology laboratory for analysis.

Culture, isolation and enrichment: Each faecal sample was cultured on D-Coccosel (bioMérieux, Marcy-l’Étoile, France) and Slanetz–Bartley agar (bioMérieux) and incubated at 37 °C for 18–24 h. After incubation, the selection of individual suspected colonies was made according to the colour and morphology. Black colonies on D-Coccosel (bioMérieux) and white colonies on Slanetz–Bartley (bioMérieux) were grown on an enrichment medium at 37 °C for 18–24 h.

Biochemical identification: The characteristic colonies obtained were identified by biochemical tests (Gram stain, catalase and a coagulase test) and API Strep strips (bioMérieux). After identification, the bacteria were stored in phosphate buffered saline (PBS)/glycerol (70/30%) at −80 °C.

Molecular identification: DNA was extracted using the boiling method described by Peng et al. [48]. The DNA extracts obtained were quantified using a NanoDrop (Nanovue plus, UK). The genus and species identifications were performed by a PCR simplex and multiplex amplification of a conserved sequence targeting the *tuf* (elongation factor) and *sodA* (superoxide oxidase) genes (Table 1)**.** The PCR mix for the genus contained 3 µL of a DNA template and 17 µL of a reaction mixture consisting of 1 X buffer, 0.2 mM dNTPs, 2.5 mM MgCl_2_, 0.2 mM of each primer, nuclease-free water and 0.5 U/mL Taq polymerase (Thermo Fischer Science, Waltham, MA, USA) for a final volume of 20 µL/tube. The PCR steps were 3 min of initial denaturation at 95 °C followed by 30 cycles of denaturation at 95 °C for 30 s, hybridisation at 55 °C for 30 s, elongation at 72 °C for 60 s and a final elongation at 72 °C for 7 min.

For the *Enterococcus* species, the PCR mix contained 5 µL of the DNA template and 25 µL of the reaction mixture consisting of 1 X buffer, 0.4 mM dNTPs, 4 mM MgCl_2_, 0.4 mM of each primer, nuclease-free water and 0.5 U/mL Taq polymerase. The PCR steps were 3 min of initial denaturation at 95 °C followed by 40 cycles of denaturation at 95 °C for 30 s, hybridisation at 52 °C for 30 s, elongation at 72 °C for 60 s and a final elongation at 72 °C for 7 min. All genomic DNA extracts were amplified using a T100TM thermal cycler (BIO RAD, Singapore) followed by a 2% agarose migration in Tris-Borate-EDTA (TBE) and visualisation under a UV light (VILBER LOURMAT, Marne La Vallée, France). Although controls were not included, the expected gel bands reported in previous publications [49,50] were ascertained through electrophoresis. The authors also ensured aseptic conditions to avoid any form of contamination. After confirming the presence of the required PCR products on the gel, the amplicons were sent to Macrogen (Amsterdam, Pays-Bas) for Sanger sequencing. The analysis and identification of these sequences were performed online using the BLAST program available from the National Center for Biotechnology Information webpage (http://www.ncbi.nlm.nih.gov).

**Table 1:** Primer sequences for *Enterococcus* spp, *E. faecium* and *E. hirae*.

Species	Target Gene	Sequence (5′-3′)	Cycles	Product Size (bp)	Reference
*Enterococcus* spp	*tuf*	TACTGACAAACCATTCATGATG AACTTCGTCACCAACGCGAAC	30	112	[50]
*E. faecium*	*sodA*	GAAAAACAATAGAAGAATTAT	40	187	[50]
		TGCTTTTTTGAATTCTTCTTTA			
*E. hirae*	*sodA*	CTTTCTGATATGGATGCTGTC	40	215	[50]
		TAAATTCTTCCTTAAATGTTG			

Antibiotic susceptibility testing: Antibiotic susceptibility testing was performed using Kirby–Bauer disk-diffusion [51]. The antibiotics tested were chosen according to those used on farms and those recommended by the Clinical Laboratory Standards Institute (CLSI) (CLSI, 2014). The antibiotics tested were tetracycline (TET, 30 µg), vancomycin (VAN, 5 µg), teicoplanin (TEI, 30 µg) and rifampicin (RIF, 5 µg).

Molecular identification of *tet(M)*: The *tet(M)* gene in the isolates was detected by a PCR amplification using the primers 5′-GTTAAATAGTGTTCTTGGAG-3′ and 5′-CTAAGATATGGCTAACAA-3′ as described by Fazzon et al. [47]. The PCR mix was composed of 1 µL of DNA and 44 µL of a reaction mixture consisting of 1 X buffer, 0.2 mM dNTP, 4 mM MgCl2, 0.2 mM primers, nuclease-free water and 0.5 U/mL Taq polymerase. The amplification was performed at 94 °C for 4 min followed by 35 cycles at 94 °C for 1 min, 58 °C for 1 min, 72 °C for 1 min and a final extension at 72 °C for 5 min. All genomic DNA extracts were amplified, migrated and visualised as previously described. The analysis and the identification of these sequences were performed online using the BLAST program available from the National Center for Biotechnology Information webpage (http://www.ncbi.nlm.nih.gov).

Phylogenetic analysis: A phylogenetic tree was constructed based on the Sanger sequencing of the *sodA* gene of *E. hirae* and *E. faecium*. This tree was constructed using 10 randomly selected sequences and 13 reference sequences from various origins (clinical, human and fish isolates) and one *E. hirae* ATCC 9790. A phylogenetic tree was constructed to understand the evolutionary relationships among the different antibiotic-resistant *E. hirae* and *E. faecium* strains. The maximum likelihood method after an alignment with ClustalW (v. 1.8.1 in BioEdit v. 7.0.9.0 software, Ibis Therapeutics, Carlsbad, CA, USA) was used. The final phylogenetic tree construction was obtained using MEGA 6 [52] for the nearest neighbour + subtree pruning (SPR) branch exchange and 100 bootstrap replicates.

Data analysis: The statistical analyses were performed using R software (version Ri386 3.5.1, Foundation for Statistical Computing, Vienna, Austria). The chi-squared test (χ2) was used to test the relationship between the parameters (local and exotic breeds, type of farms, species of animals sampled, antibiotic used) and we considered the differences significant at *p* < 0.05. The sampling map was constructed using the software QGIS3 v3.18; the data source was diva-gis.org.

## 5. Conclusions

No other studies have characterised antibiotic resistance in relation to antibiotic consumption by livestock in Gabon. Our work allowed us to describe the antibiotics used in cattle and their impact on the emergence of resistance in *E. faecium* and *E. hirae* species in Gabon. Prophylaxis was the most used method to treat animals. The most commonly used antibiotic on the farms was tetracycline. *E. faecium* and *E. hirae* species showed high frequencies of resistance to tetracycline, rifampicin and vancomycin. Tetracycline resistance is related to its use in livestock. In contrast, resistance to rifampicin and vancomycin is thought to be related to other sources not explored in this study. Studies of antibiotic resistance in wastewater could help determine the origin. The characterisation of other enterococcal species associated with their respective resistance would provide additional information on the resistance present on farms. Antibiotic stewardship committees should be established and the education of farmers on antibiotic use should be implemented to avoid the emergence of antibiotic resistance that could be transmitted to humans.

## Data Availability

Not applicable.

## Supplementary Materials

The following are available online at https://www.mdpi.com/article/10.3390/antibiotics11020224/s1, File S1: questionnary table.
