# Supplementary material for: The Antibiotics Used in Livestock and Their Impact on Resistance in Enterococcus faecium and Enterococcus hirae on Farms in Gabon
Source: Antibiotics (Basel). 2022 Feb 10;11(2):224. doi: 10.3390/antibiotics11020224 (PMC8868485; doi:10.3390/antibiotics11020224)
Supplement: Supplementary file 1 [file antibiotics-11-00224-s001.zip › antibiotics-1301790-supplementary.pdf]

Questionnaire étude

2018-2019 - CIRMF

Désiré Enquete

Donnée Social

1. quel est le nom de votre ferme?

2. Quel est le nom de l'éleveur ?

3. Quel est le sexe de l'éleveur?
- ○ 1. Feminin ○ 2. Masculin

4. Dans quel province se situe votre élevage?
- ○ 1. Haut-Ogoouée ○ 2. Ogoouée-Maritime
- ○ 3. Ogoouée-lolo ○ 4. Ogoouée-Ivindo
- ○ 5. Nyanga ○ 6. Ngounié
- ○ 7. Estuaire ○ 8. Moyen-Ogoouée
- ○ 9. Woleu-Ntem

5. Avez-vous eu une formation en élevage?
- ○ 1. oui ○ 2. non

Caractéristique de l'élevage

6. Quel type d'élevage avez-vous?
- ○ 1. Poules pondeuses ○ 2. bovins ○ 3. porcs
- ○ 4. petits ruminants ○ 5. autres

7. Quel est l'origine de vos animaux?
- ☐ 1. locale ☐ 2. importée

Vous pouvez cocher plusieurs cases.

8. Quel nombre d'animaux compte votre exploitation?
- ○ 1. 1-10 ○ 2. 11-20 ○ 3. 21-40
- ○ 4. 41-60 ○ 5. 61-80 ○ 6. 81-100
- ○ 7. 101-150 ○ 8. 151-200 ○ 9. 201-250
- ○ 10. 251-300 ○ 11. 301-350 ○ 12. 351-400
- ○ 13. 401-450 ○ 14. 451-500 ○ 15. 501-550
- ○ 16. 551-600 ○ 17. 601-650 ○ 18. 651-700
- ○ 19. 751-800 ○ 20. 801-850 ○ 21. 851-900
- ○ 22. 901-950 ○ 23. 951-1000 ○ 24. 1001-1100
- ○ 25. 1101-1200 ○ 26. 1201-1300 ○ 27. 1301-1400
- ○ 28. 1401-1500 ○ 29. 1501-1600 ○ 30. 1601-1700
- ○ 31. 1701-1800 ○ 32. 1801-1900 ○ 33. 1901-2000
- ○ 34. 2001-...

9. Dans quels conditions élevées vous les animaux ?
- ○ 1. Cloisonnement ○ 2. Air/libre
- ○ 3. cloisonnement/air libre

10. Quel est le type d'alimentation pour vos animaux?
- ☐ 1. SMAG ☐ 2. Dechets ☐ 3. Paturage
- ☐ 4. Broutage ☐ 5. autres

Vous pouvez cocher plusieurs cases (3 au maximum).

11. Abattez-vous les animaux ?
- ○ 1. OUI ○ 2. NON

12. Quel instrument utilisez-vous pour abattre les animaux ?
- ☐ 1. Machette ☐ 2. Couteau ☐ 3. Hache
- ☐ 4. autres instruments

Vous pouvez cocher plusieurs cases (2 au maximum).
La question n'est pertinente que si Abatage-Animaux = "OUI"

Santé des animaux

13. utilisez-vous des traitements/ médicaments?
- ○ 1. oui ○ 2. non

Aller à '21-vétérinaire' si Médicaments = "non"

14. Qu'est ce que vous utilisez pour le traitement tradditionnel?
- ☐ 1. Herbacées ☐ 2. Fruits ☐ 3. Potion ☐ 4. autres

Vous pouvez cocher plusieurs cases (2 au maximum).

15. Quel type de medecine utilisez vous?
- ○ 1. Tradditionnelle ○ 2. Moderne

16. Quel est votre fournisseur d'antibiotique?
- ○ 1. Gabonaise de chimie ○ 2. Point vert ○ 3. autres

17. Quel type médicament utilisez-vous?
- ☐ 1. Oxytétracycline ☐ 2. Vitamec ☐ 3. tétracolivic
- ☐ 4. Tylopharm ☐ 5. Amin total ☐ 6. Primadex
- ☐ 7. Vitajec ☐ 8. Sulferan4 ☐ 9. Norfloxan
- ☐ 10. alphaseryl ☐ 11. Colidox ☐ 12. Amprolium
- ☐ 13. Levalap ☐ 14. Epaturil ☐ 15. autres

Vous pouvez cocher plusieurs cases (10 au maximum).

18. Administrez-vous vous même les antibiotiques?
- ○ 1. oui ○ 2. non

19. Quel est la voie d'administration utilisée?
- ☐ 1. Orale ☐ 2. Injectable

Vous pouvez cocher plusieurs cases.

20. Quel est le type d'administration utilisée?

- ☐ 1. Prophylaxie
- ☐ 2. Thérapeutique
- ☐ 3. Methaphylaxie

Vous pouvez cocher plusieurs cases (2 au maximum).

21. Faites-vous appel à un vétérinaire?

- ○ 1. Jamais
- ○ 2. Occasionellement
- ○ 3. Fréquement
- ○ 4. Toujours

22. Observez-vous des cas de maladies?

- ○ 1. Jamais
- ○ 2. rarement
- ○ 3. fréquement
- ○ 4. toujours

23. Description des symptômes observées?

24. Avez-vous observez des Echecs Thérapeutiques?

- ○ 1. Non
- ○ 2. Oui

25. Comment procédez-vous en cas d'échec thérapeutique?

Protection et Hygiéne

26. Y'a t-il de la mortalité dans le troupeau?

- ○ 1. oui
- ○ 2. non

Aller à '30-Protection' si Mortalité = "oui"
La question n'est pertinente que si Mortalité = "oui"

27. Quel est la cause de la vos mortalités

- ☐ 1. maladie
- ☐ 2. écrasement
- ☐ 3. accident (voiture, camion)
- ☐ 4. malnutrition/ carence

Vous pouvez cocher plusieurs cases (3 au maximum).

28. Nombre de cas observé?

- ○ 1. 1-5
- ○ 2. 6-10
- ○ 3. 11-15
- ○ 4. 16-20
- ○ 5. 21-25
- ○ 6. 26-30
- ○ 7. 31-35
- ○ 8. 36-40
- ○ 9. 41-45
- ○ 10. 46-50
- ○ 11. 51-55
- ○ 12. 56-60
- ○ 13. 61-65
- ○ 14. 66-70
- ○ 15. 71-75
- ○ 16. 76+...
- ○ 17. Pas de dénombrement

La question n'est pertinente que si Mortalité = "oui"

29. Que faites-vous des animaux mort?

- ○ 1. dans l'environnement
- ○ 2. enterrement
- ○ 3. incinération
- ○ 4. consommation
- ○ 5. autres

30. Est-ce que vous vous protégez lors du contact avec les animaux?

- ○ 1. oui
- ○ 2. non

La question n'est pertinente que si Protection = "oui"

31. Quel sont vos moyens de protection?

- ☐ 1. blouse
- ☐ 2. combinaison de protection
- ☐ 3. gants
- ☐ 4. bottes
- ☐ 5. contrôle sanitaire auprès du CTA
- ☐ 6. Masque respiratoire
- ☐ 7. Suivi Médical pendant 3 mois

Vous pouvez cocher plusieurs cases (4 au maximum).
